# Ortho­rhom­bic polymorph of 4-[(1*H*-benzimidazol-1-yl)meth­yl]benzoic acid

**DOI:** 10.1107/S1600536811042838

**Published:** 2011-10-22

**Authors:** Hai-Wei Kuai, Xiao-Chun Cheng

**Affiliations:** a Faculty of Life Science and Chemical Engineering, Huaiyin Institute of Technology, Huaian 223003, People’s Republic of China

## Abstract

We reported recently the first polymorph of the title compound [Kuai & Cheng (2011*a* ▶). *Acta Cryst.*, **E67**, o2787]. A second polymorph of the title compound, C_15_H_12_N_2_O_2_, was unexpectedly obtained by the hydro­thermal reaction of the title compound with manganese chloride in the presence of potassium hydroxide at 413 K. The benzimidazole ring system is almost planar, with a maximum deviation from the mean plane of 0.015 (2) Å. The benzimidazole and benzene rings are inclined at a dihedral angle of 79.00 (1)°. In the crystal, adjacent mol­ecules are connected through O—H⋯N hydrogen bonds into a one-dimensional chain along the [001] direction.

## Related literature

For the synthesis of 4-[(1*H*-benzo[*d*]imidazol-1-yl)meth­yl]­benzoic acid, see: Hua *et al.* (2010 ▶). For two other polymorphs of the title compound, see: Kuai & Cheng (2011*a* ▶,*b* ▶). For related structures, see Das & Bharadwaj (2009 ▶).
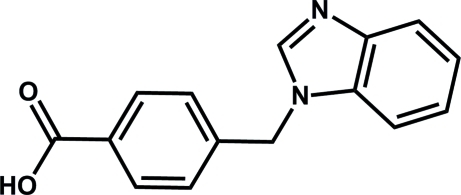


## Experimental

### 

#### Crystal data

C_15_H_12_N_2_O_2_
*M* *_r_* = 252.27
Orthorhombic, 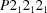

*a* = 5.6969 (15) Å
*b* = 12.657 (3) Å
*c* = 17.604 (5) Å
*V* = 1269.4 (6) Å^3^
*Z* = 4
Mo *K*α radiation
μ = 0.09 mm^−1^
*T* = 293 K
0.30 × 0.18 × 0.18 mm

#### Data collection

Bruker APEXII CCD diffractometer
Absorption correction: multi-scan (*SADABS*; Sheldrick, 1996 ▶) *T*_min_ = 0.974, *T*_max_ = 0.984
7948 measured reflections
1786 independent reflections
1313 reflections with *I* > 2σ(*I*)
*R*_int_ = 0.040

#### Refinement

*R*[*F* ^2^ > 2σ(*F* ^2^)] = 0.039
*wR*(*F* ^2^) = 0.089
*S* = 0.99
1786 reflections
166 parameters
H-atom parameters constrained
Δρ_max_ = 0.11 e Å^−3^
Δρ_min_ = −0.15 e Å^−3^

### 

Data collection: *APEX2* (Bruker, 2008 ▶); cell refinement: *SAINT* (Bruker, 2008 ▶); data reduction: *SAINT*; program(s) used to solve structure: *SHELXS97* (Sheldrick, 2008 ▶); program(s) used to refine structure: *SHELXL97* (Sheldrick, 2008 ▶); molecular graphics: *DIAMOND* (Brandenburg, 2000 ▶); software used to prepare material for publication: *SHELXTL* (Sheldrick, 2008 ▶).

## Supplementary Material

Crystal structure: contains datablock(s) I, global. DOI: 10.1107/S1600536811042838/aa2024sup1.cif

Structure factors: contains datablock(s) I. DOI: 10.1107/S1600536811042838/aa2024Isup2.hkl

Supplementary material file. DOI: 10.1107/S1600536811042838/aa2024Isup4.cdx

Supplementary material file. DOI: 10.1107/S1600536811042838/aa2024Isup4.cml

Additional supplementary materials:  crystallographic information; 3D view; checkCIF report

## Figures and Tables

**Table 1:** Hydrogen-bond geometry (Å, °)

*D*—H⋯*A*	*D*—H	H⋯*A*	*D*⋯*A*	*D*—H⋯*A*
O1—H12⋯N12^i^	0.82	1.84	2.649 (3)	168

Symmetry code: (i) 
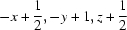
.

**Fig. 1.**
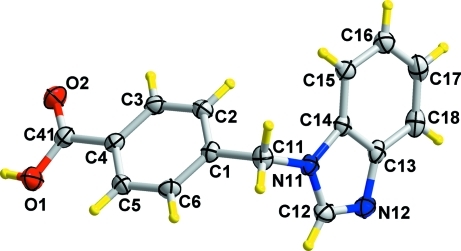
: The crystal structure of (I) showing 30% probability displacement ellipsoids.

**Fig. 2.**
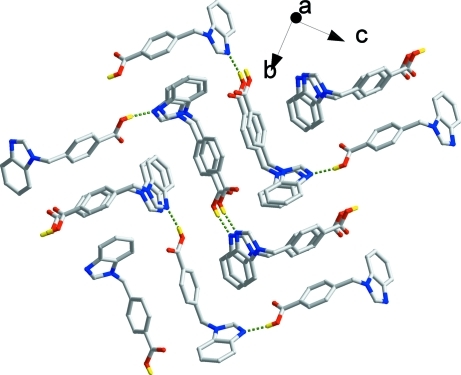
: The packing diagram of (I). Hydrogen bonds are shown as dashed lines.

## supplementary crystallographic information

### Comment

The title compound, C_15_H_12_N_2_O_2_ (I), is usually regarded as an excellent candidate for building block in molecular self-assembly engineering due to its variable conformation and coordination modes (Das & Bharadwaj, 2009). During assembly of a coordination polymer, we accidentally obtained three polymorphs of (I), which can be proved by different unit-cell parameters and space groups. Here, we are introducing one of them. The single crystals of (I) were accidentally obtained by the hydrothermal reaction at 413 K of (I) with manganese chloride in the presence of potassium hydroxide as alkaline medium for the deprotonation. As shown in Fig. 1, the asymmetric unit of (I) consists of only one molecule. Interestingly, though crystallizing from alkaline solution, (I) remains the intact carboxylic group in the crystal structure. The flexible benzimidazolyl arm is apt to rotate. As a result, the benzimidazolyl ring and central benzene rings are inclined at a dihedral angle of 79.00 (1) °; The torsion angles N11—C11—C1—C2 and N11—C11—C1—C6 are -61.8 (2) ° and 118.0 (2) °, respectively. Adjacent molecules are connected through O—H···N hydrogen bonds into a one-dimensional chain along [001] direction (Fig. 2, Table 1).

### Experimental

Reaction mixture of MnCl_2_ (21.5 mg, 0.1 mmol), 4-((1*H*-benzo[*d*]imidazol-1-yl)methyl)benzoic acid (25.2 mg, 0.1 mmol) and KOH (5.61 mg, 0.1 mmol) in 10 ml H_2_O was sealed in a 16 ml Teflon-lined stainless steel container and heated to 413 K for 3 days. After cooling to the room temperature, colorless block crystals of the title compound were obtained.

### Refinement

All hydrogen atoms were located in geometrically idealized positions and constrained to ride on their parent atoms, with C—H = 0.93–0.97, O—H = 0.82 Å and *U*_iso_(H) = 1.2*U*_eq_(C, or O). Absolute structure can not be determined in this case because of no heavy atoms present. Friedel-pair data are merged with the MERG 3 instruction. The number of Friedel pairs is 1229.

### Crystal data

**Table:**

C_15_H_12_N_2_O_2_	*F*(000) = 528
*M**_r_* = 252.27	*D*_x_ = 1.320 Mg m^−^^3^
Orthorhombic, *P*2_1_2_1_2_1_	Mo *K*α radiation, λ = 0.71073 Å
Hall symbol: P 2ac 2ab	Cell parameters from 1326 reflections
*a* = 5.6969 (15) Å	θ = 2.3–19.9°
*b* = 12.657 (3) Å	µ = 0.09 mm^−^^1^
*c* = 17.604 (5) Å	*T* = 293 K
*V* = 1269.4 (6) Å^3^	Block, colorless
*Z* = 4	0.30 × 0.18 × 0.18 mm

### Data collection

**Table:**

Bruker APEXII CCD diffractometer	1786 independent reflections
Radiation source: fine-focus sealed tube	1313 reflections with *I* > 2σ(*I*)
graphite	*R*_int_ = 0.040
φ and ω scans	θ_max_ = 28.0°, θ_min_ = 2.0°
Absorption correction: multi-scan (*SADABS*; Sheldrick, 1996)	*h* = −7→7
*T*_min_ = 0.974, *T*_max_ = 0.984	*k* = −14→16
7948 measured reflections	*l* = −23→20

### Refinement

**Table:**

Refinement on *F*^2^	Primary atom site location: structure-invariant direct methods
Least-squares matrix: full	Secondary atom site location: difference Fourier map
*R*[*F*^2^ > 2σ(*F*^2^)] = 0.039	Hydrogen site location: inferred from neighbouring sites
*wR*(*F*^2^) = 0.089	H-atom parameters constrained
*S* = 0.99	*w* = 1/[σ^2^(*F*_o_^2^) + (0.0455*P*)^2^] where *P* = (*F*_o_^2^ + 2*F*_c_^2^)/3
1786 reflections	(Δ/σ)_max_ < 0.001
166 parameters	Δρ_max_ = 0.11 e Å^−^^3^
0 restraints	Δρ_min_ = −0.15 e Å^−^^3^

### Special details

**Table:**

Geometry. All s.u.'s (except the s.u. in the dihedral angle between two l.s. planes) are estimated using the full covariance matrix. The cell s.u.'s are taken into account individually in the estimation of s.u.'s in distances, angles and torsion angles; correlations between s.u.'s in cell parameters are only used when they are defined by crystal symmetry. An approximate (isotropic) treatment of cell s.u.'s is used for estimating s.u.'s involving l.s. planes.
Refinement. Refinement of *F*^2^ against ALL reflections. The weighted *R*-factor *wR* and goodness of fit *S* are based on *F*^2^, conventional *R*-factors *R* are based on *F*, with *F* set to zero for negative *F*^2^. The threshold expression of *F*^2^ > σ(*F*^2^) is used only for calculating *R*-factors(gt) *etc*. and is not relevant to the choice of reflections for refinement. *R*-factors based on *F*^2^ are statistically about twice as large as those based on *F*, and *R*- factors based on ALL data will be even larger. Absolute structure can not be determined in this case because of no heavy atoms present. Friedel-pair data are merged with the MERG 3 instruction. The number of Friedel pairs is 1229.

### Fractional atomic coordinates and isotropic or equivalent isotropic displacement parameters (Å^2^)

**Table:**

	*x*	*y*	*z*	*U*_iso_*/*U*_eq_	
O1	0.3871 (4)	0.37836 (13)	1.03801 (9)	0.0731 (6)	
H12	0.3280	0.3376	1.0688	0.088*	
O2	0.0975 (4)	0.48320 (13)	1.07621 (9)	0.0650 (5)	
N11	0.4456 (3)	0.78681 (13)	0.75703 (9)	0.0412 (4)	
N12	0.2556 (4)	0.74457 (14)	0.65069 (10)	0.053	
C4	0.3625 (4)	0.54619 (16)	0.98240 (11)	0.0390 (5)	
C11	0.6074 (4)	0.77548 (17)	0.82087 (11)	0.0451 (5)	
H6	0.6262	0.8434	0.8456	0.054*	
H5	0.7598	0.7537	0.8019	0.054*	
C13	0.1428 (4)	0.83025 (16)	0.68400 (11)	0.0417 (5)	
C14	0.2622 (4)	0.85805 (15)	0.75045 (11)	0.0372 (5)	
C6	0.6520 (4)	0.60602 (17)	0.89316 (11)	0.0458 (5)	
H4	0.7940	0.5954	0.8682	0.055*	
C5	0.5733 (4)	0.53174 (17)	0.94491 (11)	0.0473 (6)	
H3	0.6627	0.4717	0.9545	0.057*	
C41	0.2678 (5)	0.46665 (17)	1.03690 (11)	0.0472 (6)	
C15	0.1919 (4)	0.94228 (17)	0.79536 (12)	0.0462 (6)	
H8	0.2725	0.9610	0.8393	0.055*	
C1	0.5221 (4)	0.69567 (16)	0.87823 (11)	0.0374 (5)	
C17	−0.1262 (5)	0.9687 (2)	0.70573 (13)	0.0572 (7)	
H10	−0.2587	1.0071	0.6920	0.069*	
C12	0.4308 (5)	0.72183 (17)	0.69610 (12)	0.0510 (6)	
H7	0.5348	0.6665	0.6873	0.061*	
C16	−0.0036 (5)	0.99675 (19)	0.77139 (13)	0.0557 (6)	
H9	−0.0557	1.0541	0.7998	0.067*	
C2	0.3115 (4)	0.71014 (16)	0.91701 (11)	0.0437 (5)	
H1	0.2224	0.7704	0.9081	0.052*	
C3	0.2344 (4)	0.63641 (15)	0.96819 (11)	0.0426 (5)	
H2	0.0936	0.6474	0.9937	0.051*	
C18	−0.0555 (4)	0.88605 (18)	0.66126 (13)	0.0526 (6)	
H11	−0.1370	0.8678	0.6174	0.063*	

### Atomic displacement parameters (Å^2^)

**Table:**

	*U*^11^	*U*^22^	*U*^33^	*U*^12^	*U*^13^	*U*^23^
O1	0.0997 (15)	0.0514 (10)	0.0681 (10)	0.0157 (11)	0.0362 (11)	0.0196 (8)
O2	0.0673 (13)	0.0707 (11)	0.0571 (9)	0.0034 (10)	0.0240 (10)	0.0109 (8)
N11	0.0449 (11)	0.0390 (9)	0.0396 (9)	−0.0030 (9)	−0.0010 (8)	0.0024 (8)
N12	0.072	0.044	0.042	−0.007	−0.005	−0.002
C4	0.0435 (13)	0.0422 (11)	0.0313 (10)	0.0001 (10)	0.0020 (10)	−0.0025 (8)
C11	0.0395 (13)	0.0473 (12)	0.0484 (12)	−0.0053 (11)	−0.0046 (11)	0.0069 (10)
C13	0.0494 (14)	0.0382 (11)	0.0375 (10)	−0.0108 (10)	−0.0017 (10)	0.0050 (9)
C14	0.0382 (12)	0.0354 (10)	0.0379 (10)	−0.0067 (9)	0.0022 (10)	0.0054 (9)
C6	0.0354 (13)	0.0556 (13)	0.0462 (12)	0.0063 (11)	0.0079 (11)	0.0022 (11)
C5	0.0509 (15)	0.0467 (12)	0.0442 (12)	0.0138 (11)	0.0048 (11)	0.0069 (10)
C41	0.0569 (15)	0.0505 (14)	0.0341 (10)	0.0001 (12)	0.0032 (12)	0.0005 (10)
C15	0.0542 (16)	0.0442 (12)	0.0404 (11)	−0.0009 (11)	−0.0001 (11)	0.0002 (10)
C1	0.0360 (12)	0.0400 (11)	0.0363 (10)	−0.0036 (9)	−0.0045 (9)	−0.0012 (9)
C17	0.0488 (15)	0.0614 (15)	0.0613 (15)	0.0054 (13)	−0.0005 (13)	0.0225 (13)
C12	0.0672 (17)	0.0371 (12)	0.0487 (12)	−0.0035 (12)	0.0066 (12)	−0.0026 (10)
C16	0.0621 (16)	0.0493 (13)	0.0557 (14)	0.0082 (12)	0.0102 (14)	0.0079 (11)
C2	0.0441 (14)	0.0394 (12)	0.0476 (11)	0.0064 (10)	0.0007 (11)	0.0004 (10)
C3	0.0395 (12)	0.0478 (12)	0.0403 (11)	0.0048 (10)	0.0054 (11)	−0.0039 (10)
C18	0.0535 (16)	0.0557 (14)	0.0487 (13)	−0.0134 (13)	−0.0128 (12)	0.0152 (12)

### Geometric parameters (Å, °)

**Table:**

O1—C41	1.308 (3)	C6—C1	1.380 (3)
O1—H12	0.8200	C6—C5	1.384 (3)
O2—C41	1.210 (3)	C6—H4	0.9300
N11—C12	1.354 (3)	C5—H3	0.9300
N11—C14	1.385 (3)	C15—C16	1.376 (3)
N11—C11	1.461 (3)	C15—H8	0.9300
N12—C12	1.311 (3)	C1—C2	1.392 (3)
N12—C13	1.390 (3)	C17—C18	1.367 (3)
C4—C3	1.378 (3)	C17—C16	1.396 (3)
C4—C5	1.382 (3)	C17—H10	0.9300
C4—C41	1.492 (3)	C12—H7	0.9300
C11—C1	1.509 (3)	C16—H9	0.9300
C11—H6	0.9700	C2—C3	1.369 (3)
C11—H5	0.9700	C2—H1	0.9300
C13—C18	1.391 (3)	C3—H2	0.9300
C13—C14	1.398 (3)	C18—H11	0.9300
C14—C15	1.386 (3)		
			
C41—O1—H12	109.5	O2—C41—C4	122.8 (2)
C12—N11—C14	106.40 (18)	O1—C41—C4	113.5 (2)
C12—N11—C11	126.09 (19)	C16—C15—C14	116.4 (2)
C14—N11—C11	127.18 (16)	C16—C15—H8	121.8
C12—N12—C13	105.42 (18)	C14—C15—H8	121.8
C3—C4—C5	118.88 (19)	C6—C1—C2	118.50 (19)
C3—C4—C41	119.0 (2)	C6—C1—C11	120.3 (2)
C5—C4—C41	122.1 (2)	C2—C1—C11	121.2 (2)
N11—C11—C1	112.17 (17)	C18—C17—C16	121.4 (2)
N11—C11—H6	109.2	C18—C17—H10	119.3
C1—C11—H6	109.2	C16—C17—H10	119.3
N11—C11—H5	109.2	N12—C12—N11	113.4 (2)
C1—C11—H5	109.2	N12—C12—H7	123.3
H6—C11—H5	107.9	N11—C12—H7	123.3
N12—C13—C18	130.5 (2)	C15—C16—C17	122.1 (2)
N12—C13—C14	108.94 (19)	C15—C16—H9	119.0
C18—C13—C14	120.5 (2)	C17—C16—H9	119.0
N11—C14—C15	132.13 (19)	C3—C2—C1	120.6 (2)
N11—C14—C13	105.84 (17)	C3—C2—H1	119.7
C15—C14—C13	122.0 (2)	C1—C2—H1	119.7
C1—C6—C5	120.7 (2)	C2—C3—C4	121.0 (2)
C1—C6—H4	119.7	C2—C3—H2	119.5
C5—C6—H4	119.7	C4—C3—H2	119.5
C4—C5—C6	120.4 (2)	C17—C18—C13	117.6 (2)
C4—C5—H3	119.8	C17—C18—H11	121.2
C6—C5—H3	119.8	C13—C18—H11	121.2
O2—C41—O1	123.8 (2)		
			
C12—N11—C11—C1	−80.9 (3)	N11—C14—C15—C16	−179.6 (2)
C14—N11—C11—C1	91.6 (2)	C13—C14—C15—C16	0.6 (3)
C12—N12—C13—C18	−178.9 (2)	C5—C6—C1—C2	0.7 (3)
C12—N12—C13—C14	1.0 (2)	C5—C6—C1—C11	−179.09 (19)
C12—N11—C14—C15	−179.4 (2)	N11—C11—C1—C6	118.0 (2)
C11—N11—C14—C15	7.0 (3)	N11—C11—C1—C2	−61.8 (2)
C12—N11—C14—C13	0.4 (2)	C13—N12—C12—N11	−0.7 (2)
C11—N11—C14—C13	−173.21 (18)	C14—N11—C12—N12	0.2 (2)
N12—C13—C14—N11	−0.9 (2)	C11—N11—C12—N12	173.93 (19)
C18—C13—C14—N11	179.04 (18)	C14—C15—C16—C17	0.4 (3)
N12—C13—C14—C15	179.0 (2)	C18—C17—C16—C15	−0.9 (4)
C18—C13—C14—C15	−1.1 (3)	C6—C1—C2—C3	−0.7 (3)
C3—C4—C5—C6	−1.0 (3)	C11—C1—C2—C3	179.11 (19)
C41—C4—C5—C6	178.2 (2)	C1—C2—C3—C4	−0.2 (3)
C1—C6—C5—C4	0.1 (3)	C5—C4—C3—C2	1.0 (3)
C3—C4—C41—O2	−9.2 (3)	C41—C4—C3—C2	−178.2 (2)
C5—C4—C41—O2	171.7 (2)	C16—C17—C18—C13	0.3 (3)
C3—C4—C41—O1	171.1 (2)	N12—C13—C18—C17	−179.5 (2)
C5—C4—C41—O1	−8.0 (3)	C14—C13—C18—C17	0.6 (3)

### Hydrogen-bond geometry (Å, °)

**Table:**

*D*—H···*A*	*D*—H	H···*A*	*D*···*A*	*D*—H···*A*
O1—H12···N12^i^	0.82	1.84	2.649 (3)	168.

Symmetry codes: (i) −*x*+1/2, −*y*+1, *z*+1/2.
